# Histone deacetylase inhibition with givinostat: a multi-targeted mode of action with the potential to halt the pathological cascade of Duchenne muscular dystrophy

**DOI:** 10.3389/fcell.2024.1514898

**Published:** 2025-01-06

**Authors:** A. Aartsma-Rus

**Affiliations:** Department of Human Genetics, Leiden University Medical Center (LUMC), Leiden, Netherlands

**Keywords:** dystrophinopathy, acetylation, muscle repair, myogenesis, inflammation, FAP cells, satellite cells, miRNA

## Abstract

Muscle repair and regeneration are complex processes. In Duchenne muscular dystrophy (DMD), these processes are disrupted by the loss of functional dystrophin, a key part of the transmembrane dystrophin-associated glycoprotein complex that stabilizes myofibers, indirectly leading to progressive muscle wasting, subsequent loss of ambulation, respiratory and cardiac insufficiency, and premature death. As part of the DMD pathology, histone deacetylase (HDAC) activity is constitutively increased, leading to epigenetic changes and inhibition of muscle regeneration factors, chronic inflammation, fibrosis, and adipogenesis. HDAC inhibition has consequently been investigated as a therapeutic approach for muscular dystrophies that, significantly, works independently from specific genetic mutations, making it potentially suitable for all patients with DMD. This review discusses how HDAC inhibition addresses DMD pathophysiology in a multi-targeted mode of action and summarizes the recent evidence on the rationale for HDAC inhibition with givinostat, which is now approved by the United States Food and Drug Administration for the treatment of DMD in patients aged 6 years and older.

## Introduction

In patients with Duchenne muscular dystrophy (DMD), the direct effect of the characteristic lack of dystrophin is the loss of muscle sarcolemma membrane stability, which results in DMD pathology (Mozzetta et al., 2024). DMD is characterized by progressive loss of muscle tissues, leading to loss of ambulation and the need for assisted ventilation, and, eventually, premature death in the 2–4th decade (Ryder et al., 2017; Walter and Reilich, 2017). Indirectly, the loss of dystrophin results in a cascade of pathological events in the muscle cell that include chronic inflammation and failed regeneration (Dowling et al., 2023). One pathological aspect perpetuating the pathological pathways is the increased activity of histone deacetylase (HDAC) enzymes. As such, the global inhibition of HDAC activity has received attention as a therapeutic approach for treating muscular dystrophies (Lamb, 2024). Preclinical and clinical studies have demonstrated positive effects of HDAC inhibition on multiple levels of DMD-related pathogenic events (Mozzetta et al., 2024). Pivotal phase 3 clinical trial data have recently been published for givinostat, an HDAC inhibitor that was investigated in ambulant boys aged 6 years and older with DMD (Mercuri et al., 2024). In this multicenter, randomized trial, givinostat significantly delayed DMD disease progression compared with placebo and had a positive risk/benefit profile. Givinostat has recently been approved by the US Food and Drug Administration for the treatment of DMD in patients aged 6 years and older (Lamb, 2024; United States Food and Drug Administration, 2024), and evaluation by the European Medicines Agency is ongoing (Duchenne UK, 2023). The purpose of this narrative review is to elaborate on how HDAC inhibition addresses DMD pathophysiology in a multi-targeted mode of action and to summarize the recent evidence on the rationale for HDAC inhibition with givinostat.

## Dystrophin and DMD

DMD results from mutations in the *DMD* gene encoding dystrophin, the largest known human gene (Aartsma-Rus et al., 2016; Okubo et al., 2017). So far, over 7,000 mutations in the *DMD* gene have been identified (Aartsma-Rus et al., 2016; Bladen et al., 2015). These mutations result in the absence of functional dystrophin (Blake et al., 2002; Hoffman et al., 1987).

Dystrophin has a mechanical, stabilizing function in skeletal muscle fibers by connecting the cytoskeleton—part of the contractile machinery—to the connective tissue surrounding each muscle fiber. Specifically, dystrophin binds to F-actin in the cytoskeleton and to a part of the transmembrane dystrophin-associated glycoprotein protein complex (DAPC) called beta-dystroglycan, which in turn binds to the connective tissue protein laminin (Mukund and Subramaniam, 2020; Wilson et al., 2022; Campbell and Kahl, 1989; Ervasti and Campbell, 1993; Guiraud et al., 2015).

## Normal muscle repair

**FIGURE 1:**
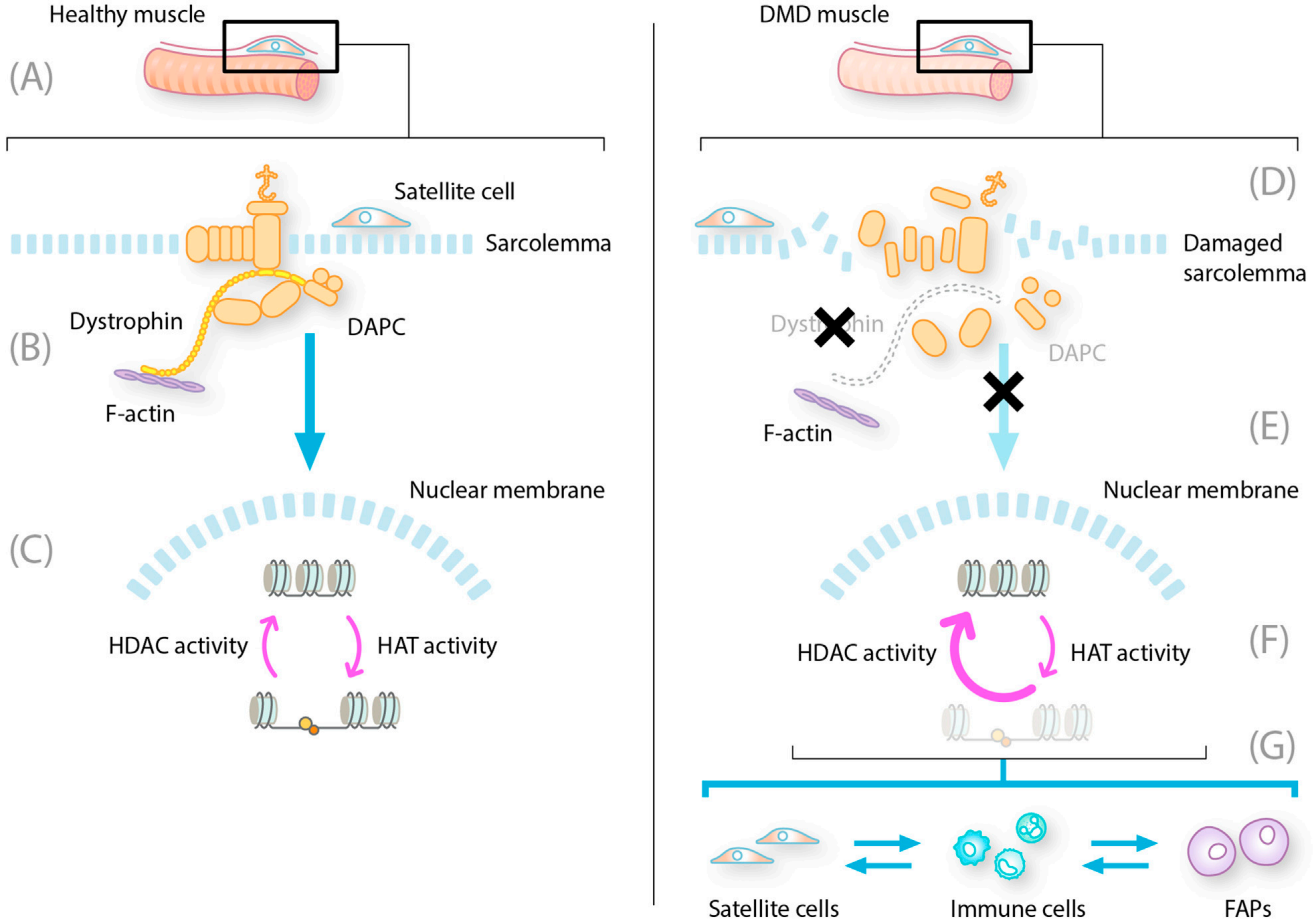
Constitutive HDAC activity contributes to dystrophic muscle pathology. DAPC, dystrophin-associated protein complex; DMD, Duchenne muscular dystrophy; FAP, fibro-adipogenic progenitor; HAT, histone acetyltransferase; HDAC, histone deacetylase; MuSC, muscle stem cell; NO, nitrous oxide; NOS, nitric oxide synthase. LEFT: healthy muscle fibers with intact dystrophin and DAPC. **(A)** MuSC and satellite cells reside on the muscle fibers ready to differentiate into new muscle fibers **(B)** Intact DAPC regulates activity of HDAC to allow translation of muscle regeneration factors **(C)** In the nucleus HDAC and HAT work in balance to regulate the expression of muscle regeneration factors RIGHT: DMD muscle with mutated dystrophin and disrupted DAPC **(D)** Absent or mutated dystrophin leads to disruption of the DAPC with multiple consequences for muscle repair **(E)** These consequences include damage to the sarcolemma leading to cytoplasmic leakage displacement of NOS and decreased levels of NO, which, in turn, lead to aberrant constitutive expression of HDACs **(F)** Increased HDAC activity results in repression of translation and transcription of muscle repair regeneration factors **(G)** The absence of gene transcription leads to changes in the production of new myofibres from satellite cells, prolongation of inflammatory phases of repair into a chronic state, and induction of FAP differentiation into fibroblasts and adipocytes.

Muscle contraction and relaxation cause stress to the muscle fibers, and damage can occur during regular activity or trauma. Muscle fibers are postmitotic, and muscle damage repair is orchestrated by satellite cells, quiescent cells that lie on top of muscle fibers, which are activated when there is damage. Upon activation, satellite cells proliferate into muscle stem cells (MuSC), which differentiate into muscle fibers to repair the damage (Figure 1) (Mukund and Subramaniam, 2020). Healthy skeletal muscle has a unique immune-privileged status, with fewer antigen-presenting cells and pro-inflammatory cells present, and no constitutive major histocompatibility complex class I (MHCI) or II (MHCII) expression, resulting in less necrosis and a lower capacity to generate abscesses (Bez Batti Angulski et al., 2023; Sciorati et al., 2016). Contraction can cause membrane damage and leakage of cytoplasmic content into the extracellular compartment. Due to skeletal muscle’s unique immune privilege, rapid membrane repair mechanisms, and membrane stability conferred by intact dystrophin, this inflammatory response is limited, controlled, and quickly resolved in healthy muscle (Sciorati et al., 2016).

Myogenesis also depends significantly on the interaction between satellite cells and their microenvironment (Mukund and Subramaniam, 2020). In healthy muscle, injury is repaired by the asymmetric division of satellite cells; the interactions of DAPC subunits are essential for this process (Chang et al., 2018; Duan et al., 2021; Dumont et al., 2015). The activation and migration of satellite cells to the injury site and their proliferation and differentiation into muscle fibers is a synchronized series of stepwise processes. First, upon muscle injury, there is an acute and transient innate immune system response. During this process, the immune system will inhibit the myogenic repair system, enabling inflammatory cells (neutrophils, eosinophils, and macrophages) to be recruited to clear the area of debris. M1 macrophages arrive first and induce a pro-inflammatory phase that, in muscle, causes the secretion of cytokines, promoting myogenic cell proliferation. In the subsequent anti-inflammatory phase, M2 macrophages facilitate myogenic differentiation, stimulating the activation, proliferation, and division of satellite cells (Blau et al., 2015; Rugowska et al., 2021; Ziemkiewicz et al., 2021). Once the damage is cleared, fibro-adipogenic progenitors (FAPs) are activated to repair the extracellular matrix. FAPs are muscle-resident multipotent mesenchymal stem cells that can differentiate into adipocytes, fibroblasts, or osteocytes. Intrinsic and extrinsic regulatory mechanisms control the activation, proliferation, cell fate decision, and clearance of FAPs (Molina et al., 2021). Finally, satellite cells are activated to proliferate and differentiate into mature muscle either by fusing with the remaining muscle fiber or by forming a new fiber within the shell of connective tissue. Many transcription factors are involved in muscle development and repair, and they have a temporal sequence of activation across various stages of myogenesis (Mukund and Subramaniam, 2020). While the differentiation of satellite cells produces new myofibers necessary for muscle repair and regeneration, the self-renewal of satellite cells is crucial for maintaining the stem cell population (Kodippili and Rudnicki, 2023).

## Failed muscle repair in DMD

The loss of functional dystrophin in DMD results in the disassembly of DAPC complexes, reduced expression levels of certain DAPC components, and the loss of the interaction between the F-actin cytoskeleton and the extracellular matrix. This leads to wide-ranging consequences, including loss of membrane and myofiber integrity, impaired muscle fiber contractile activity, contraction-induced membrane rupture, and progressive muscle degeneration (Figure 1) (Dowling et al., 2023; Kodippili and Rudnicki, 2023). The membrane leakage leads to continuous release of cytoplasmic content, including damage-associated molecular patterns that are ligands to toll-like receptors, P2RX7, and other pattern recognition receptors on muscle and adaptive immune cells (Bez Batti Angulski et al., 2023; Giordano et al., 2015; Henriques-Pons et al., 2014; Sinadinos et al., 2015). Pattern recognition receptors activate downstream signaling cascades and initiate the innate immune response, and pro-inflammatory cytokines induce the expression of MHCI and MHCII on muscle fibers, removing the immune privilege (Bez Batti Angulski et al., 2023).

Membrane leakage also results in abnormal calcium handling and associated proteolytic degradation of muscle proteins, leading to a cascade of pathological events in the muscle cell and a desynchronization of the repair processes with failure of proper myogenic repair (Dowling et al., 2023).

Compared with the cycling states in normal muscle tissue, the innate and adaptive immune system becomes chronically activated in patients with DMD, inhibiting muscle repair even at locations where the debris has been cleared. The chronic inflammatory response in muscle fibers of patients with DMD is maintained by overlapping pro- and anti-inflammatory signaling, preventing the full resolution of inflammation (Bez Batti Angulski et al., 2023). FAPs are improperly activated and persist at sites of tissue damage. Here, they produce excess connective tissue leading to fibrosis and differentiate into fibroblasts and fat cells that produce fat tissue (adiposis) (Giuliani et al., 2022). The MuSCs maintain a proliferative state, and due to the signals from inflammatory cells and FAPs, they transdifferentiate into FAPs—additionally, increased symmetric satellite cell expansion results in satellite cell hyperplasia. Fewer asymmetric cell divisions lead to low myogenic progenitor cell numbers (Kodippili and Rudnicki, 2023).

## Functions of HDACs

HDACs are evolutionarily conserved enzymes that remove acetylated groups from lysine residues in histones. This action leads to a “closed” histone structure and reduced DNA accessibility. The HDAC counterparts, histone acetylases, add acetyl groups to proteins, leading to an “open” histone structure and increased DNA accessibility for transcription factors (Eslaminejad et al., 2013; Mozzetta et al., 2024).

The combinations of histone modifications determine their overall interaction with DNA, leading to activation and/or inhibition of transcription (Eslaminejad et al., 2013). Many chromatin states are regulated and maintained in a tissue-specific way, ensuring DNA is accessible at specific times and precise locations (Mariño-Ramírez et al., 2005). Specifically, the acetylation and deacetylation of proteins influence important processes in muscle cells (Sandonà et al., 2023).

HDACs have many roles (Molinari et al., 2023; Sandonà et al., 2023); by both directly and indirectly regulating gene expression, they also control key cellular processes through the deacetylation of non-histone proteins and act as effectors in response to physiological and pathological signals (e.g., acetylation of SMADs can dampen TGF-*β* signaling by reducing SMAD phosphorylation) (Osseni et al., 2022). Genetic and pharmacological manipulations of HDACs in both *in vitro* and *in vivo* settings have emphasized their crucial role in the maintenance and adaptation of skeletal muscle metabolism (Molinari et al., 2023).

## Increased levels of HDACs in DMD

A lack of dystrophin results in HDAC hyperactivity, which exacerbates, at least in part, the pathological processes outlined above. Dystrophin, as part of the DAPC, has many other important roles in addition to providing mechanical stability to muscle fibers (Dowling et al., 2023). It is crucial for signal transduction between the internal and external environments of the muscle cell, providing a scaffold responsible for the membrane localization of signaling proteins. In health, the DAPC anchors a variety of signaling molecules to their functional sites at the sarcolemma via the syntrophin protein. One such is the enzyme nitric oxide synthase (NOS), which regulates the intramuscular generation of nitric oxide and microribonucleic acids (miRNAs) required for muscle tissue maintenance and regeneration. This is achieved through the modification of HDACs (Marrone and Shcherbata, 2011). In DMD, dystrophin loss and DAPC disassembly lead to the displacement of muscle-specific NOS. The resultant reduction in nitric oxide generation leads to an aberrant, constitutive hyperactivation of HDACs due to NO-mediated S-nitrosylation (Kodippili and Rudnicki, 2023; Marrone and Shcherbata, 2011; Sandoná et al., 2016).

## The consequences of constitutive HDAC activity

Aberrant and constitutive hyperactive HDACs in patients with DMD cause excessive acetyl group removal from histone proteins, preventing transcription of key homeostatic genes (Mozzetta et al., 2024), resulting in a decrease in the levels of myogenic miRNAs (Rugowska et al., 2021).

This has many pathological consequences for muscle damage repair. First, the immune system becomes chronically activated in the muscle (Rosenberg et al., 2015). HDACs appear to have a role in the regulation of the immune response (Licciardi and Karagiannis, 2012; Sweet et al., 2012). In the context of DMD, in which immune cells infiltrate muscles and contribute to disease pathology, the constitutive hyperactivity of HDACs affects the balance between pro-inflammatory and anti-inflammatory immune cell populations, thereby influencing disease progression (Figure 1) (Bez Batti Angulski et al., 2023; Kulthinee et al., 2022; Licciardi and Karagiannis, 2012). Specifically, HDAC hyperactivity has been associated with the suppression of regulatory T cells through the deacetylation of Foxp3 (Beier et al., 2011).

**FIGURE 2:**
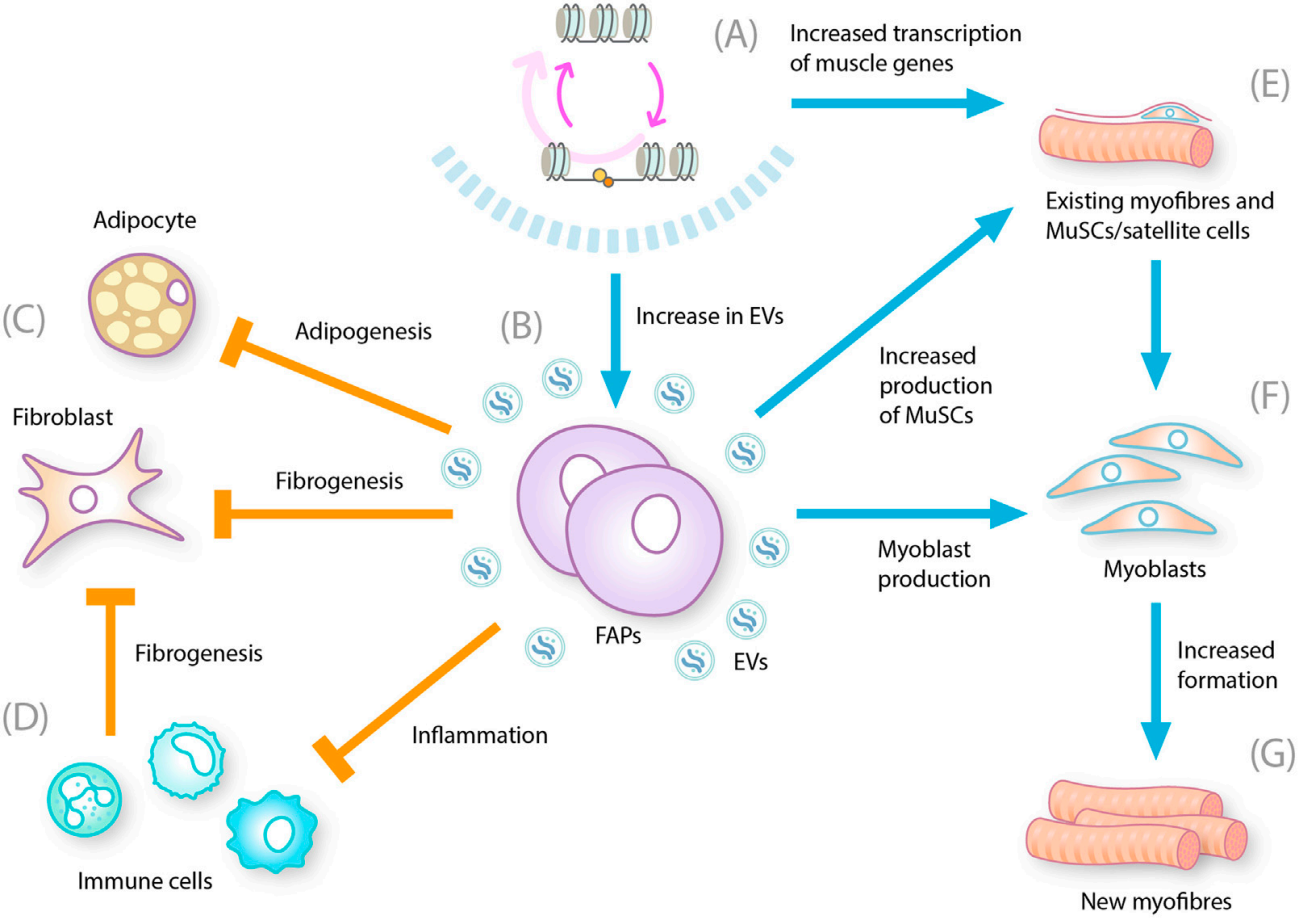
Multiple effects of HDAC inhibition on DMD-related pathogenesis. DMD, Duchenne muscular dystrophy; EV, extracellular vesicle; FAP, fibro-adipogenic progenitor; HDACi, histone deacetylase inhibitor, MuSC, muscle stem cell. HDAC inhibition leads to **(A)** Reduction in hyperacetylation of chromatin by HDAC and restoration of gene transcription **(B)** Increase in the number of EVs produced by FAPS that contain microRNAs that influence the biological processes controlling muscle regeneration, fibrogenesis, and inflammation **(C)** Decrease in the differentiation of FAP cells into adipocytes and fibroblasts **(D)** Decrease in chronic inflammation and reduction in inflammatory cytokines, which also reduces fibrosis **(E)** Increases transcription of muscle genes and decreases myofiber membrane leakage and myofiber degeneration/necrosis. Inhibits activation of TGF-*β* signaling **(F)** Increases fusion of myoblasts into differentiated myotubes **(G)** Increases formation of regenerating, center-nucleated myofibers.

Second, it results in altered FAP activity. The FAPs stall in connective tissue production mode and become fibroblasts and fat cells instead of supporting the satellite cells to differentiate and repair muscle (Figure 2) (Marrone and Shcherbata, 2011; Ren et al., 2024; Rugowska et al., 2021; Saccone et al., 2014; Sandoná et al., 2016). Evidence from a DMD mouse model (*mdx*) has revealed an HDAC-regulated network that consists of myogenic miRNAs and a chromatin remodeling complex that is able to activate the myogenic program in FAPs. HDAC-mediated repression of myogenic miRNAs is reported in dystrophic muscles (Saccone et al., 2014).

Third, activated satellite cells cannot differentiate into new fibers without FAP involvement (Figure 1). Hyperactive HDACs deacetylate faster than HATs (Hyperactive HDACs deacetylate faster than HATs acetylate), thereby reducing the activity of transcription factors and co-factors critical for myogenic differentiation, such as MyoD and MEF2 (Marrone and Shcherbata, 2011; Rugowska et al., 2021) and impairing the differentiation of satellite cells (Marrone and Shcherbata, 2011; Ren et al., 2024). This leads to ineffective muscle repair and regeneration in patients with DMD. Furthermore, the FAPs produce factors causing activated satellite cells to transdifferentiate into fibroblasts and lose muscle repair capability (Molina et al., 2021; Ren et al., 2024).

Proteins other than histones are also regulated by the addition and removal of acetyl groups, which can further exacerbate muscle damage and pathology in DMD. For example, a key regulator of fibrosis, TGF-*β*, works by triggering the addition of a phosphate group to a protein complex, SMAD, leading to fibrosis (Biernacka et al., 2011). Normally, this process is inhibited by SMAD acetylation (Osseni et al., 2022). However, with HDAC hyperactivity, these acetyl groups are actively removed, reducing the threshold for adding phosphate groups and thus further increasing fibrosis.

## HDAC inhibition in dystrophinopathy

The finding that dystrophin deficiency leads to constitutive HDAC activation in muscles has provided a rationale for investigating HDAC inhibitors such as givinostat in DMD (Lamb, 2024): given that multiple muscle damage and repair processes are exacerbated by excessive HDAC activity, inhibiting HDAC activity could improve muscle repair and alleviate DMD pathology. HDAC inhibition is expected to allow immune cells to transition from a pro-inflammatory state to a modulatory state, which would diminish the immune response and reduce the inhibition of muscle repair processes. HDAC inhibition might also direct FAPs to regain their supportive role in muscle repair and prevent their production of fat and connective tissues. Evidence suggests that FAPs exposed to HDAC inhibitors increase the extracellular vesicle levels of a subset of miRNAs that target biological processes such as regeneration, fibrosis, and inflammation (Sandonà et al., 2020). Satellite cells can also be prompted to differentiate into muscle fibers rather than remaining stalled in proliferation mode (Figure 2) (Kodippili and Rudnicki, 2023).

Exposure of *mdx* mice to HDAC inhibitors demonstrated therapeutic effects in dystrophinopathy, including histological improvement in fibrosis and inflammation, as well as enhanced muscle regeneration, decreased membrane permeability, and increased muscle strength and performance (Mozzetta et al., 2024).

## Effects of givinostat in DMD

Although HDAC inhibitors have been in development for many years and for many diseases, relatively few have been approved for use in specific indications (Mozzetta et al., 2024). HDAC inhibitors have a narrow therapeutic window; doses above a certain threshold are required for a therapeutic effect, but high doses can be associated with dose-limiting side effects (Sandonà et al., 2023).

Givinostat is an orally bioavailable, potent HDAC inhibitor (Lamb, 2024; United States Food and Drug Administration, 2024) designed to overcome the challenges observed in previous studies (Mozzetta et al., 2024). Unlike other HDAC inhibitors, givinostat has shown efficacy at dosing levels that are generally tolerated (Lamb, 2024; Mercuri et al., 2024).

On a molecular level, HDAC inhibition by givinostat leads to a cascade of changes in gene expression, protein function, and cellular processes. Specifically, one effect of HDAC inhibition by givinostat leads to hyperacetylation of histones, resulting in a more open and relaxed chromatin structure. Consequently, transcription factors and other regulatory proteins can more easily access DNA, enhancing the transcription of genes involved in muscle repair and anti-inflammatory responses. The effects of this inhibition by givinostat have been consistently demonstrated throughout a defined preclinical and clinical program.

## Preclinical evidence of givinostat efficacy

The effect of givinostat on myogenic miRNAs has been investigated in *mdx* mice. In untreated *mdx* versus wild-type mice, 120 miRNAs were found to be significantly upregulated and 66 were found to be significantly downregulated (Licandro et al., 2021), and correlations noted with patients with DMD (Cacchiarelli et al., 2011; Licandro et al., 2021). Furthermore, specific miRNAs have been shown to correlate with DMD pathology. In *mdx* mice, givinostat was shown to induce miRNAs (miR-449a-5p and miR-92b-3p) that are known to be linked to stem cell pluripotency and are reduced in patients with heart failure (Licandro et al., 2021). Both *in vitro* and *ex vivo*, givinostat restored human DMD FAP ability to support MuSC differentiation into multinucleated myotubes, indicating that additional events resulting from DAPC disassembly can be dysregulated in DMD MuSCs (Sandonà et al., 2020). The muscle regeneration factor, miR-206, is repressed in DMD MuSCs (Cacchiarelli et al., 2011); and could be compensated by FAP-derived EVs-miR-206 to restore MuSC ability to regenerate dystrophic muscle (Sandonà et al., 2020).

Histopathological analysis also demonstrated improvements with givinostat treatment. Givinostat significantly reduced fibrosis by up to 30%–40% in *mdx* mice compared with healthy controls and reduced inflammation (Consalvi et al., 2013). In another study, givinostat also reduced fibrosis, necrosis, and fat replacement in skeletal muscle tissue in *mdx* mice (Licandro et al., 2021). Fibrosis, often regarded as the most detrimental consequence of disease progression in DMD mouse models, arises from complex interactions between resident cell types and inflammatory infiltrates. The myeloperoxidase (MPO) enzyme produced by neutrophils, monocytes, and macrophages serves as a marker for quantifying inflammation linked to muscle degeneration in muscular dystrophies. Significantly reduced MPO activity was observed in the muscles of *mdx* mice treated with givinostat compared to those treated with a vehicle control. These results were also supported by functional improvements in *mdx* mice, where givinostat treatment resulted in muscle regeneration. Histological images showed that givinostat increased muscle fiber diameter (measured as myofiber cross-sectional area) in *mdx* mice compared to control (Consalvi et al., 2013). Givinostat treatment resulted in increased muscle strength, demonstrated by dose-dependent improvements in the grip test (Licandro et al., 2021). Improvements were also seen in the treadmill exhaustion test, both in terms of distance covered and time to exhaustion (Consalvi et al., 2013; Licandro et al., 2021).

## First-in-human evidence of HDAC inhibition by givinostat

Orally administered givinostat 50 or 100 mg transiently reduced the *in vitro* production of pro-inflammatory cytokines (while not affecting anti-inflammatory cytokines) in a phase 1 trial in healthy males. After seven daily doses of givinostat 200 mg, the reductions in cytokine production were generally similar to those following the initial dose (Furlan et al., 2011).

## Clinical evidence of givinostat efficacy

In an open-label, two-part, phase 2 study, the histological effects of givinostat were analyzed in 20 ambulant boys with DMD (Bettica et al., 2016). The histological effect of givinostat treatment was confirmed, with a significant increase in muscle tissue and reductions in fibrosis, tissue necrosis, and fatty replacement.

Givinostat was subsequently shown to slow DMD progression in a placebo-controlled phase 3 trial, in which both groups continued to receive corticosteroids. The results indicated that over 18 months of treatment, givinostat significantly improved muscle function and strength compared with the placebo group. It effectively slowed the progression of muscle degeneration in the participants. The primary endpoint, change in four-stair climb from baseline, was met. All secondary endpoints were in favor of givinostat, including other timed function tests, the North Star Ambulatory Assessment and muscle strength. Givinostat reduced fat infiltration in the vastus lateralis in patients with DMD by 30%. Magnetic resonance spectroscopy evidence suggested less fat infiltration in the vastus lateralis at 72 weeks with givinostat compared to control group (LSM difference in fat fraction −2.92% [–5.64 to −0.20] (Mercuri et al., 2024). These findings are consistent with those observed in the givinostat pre-clinical studies (Consalvi et al., 2013; Licandro et al., 2021) and phase 2 clinical trial (Bettica et al., 2016).

In terms of safety, givinostat was generally well tolerated. The most common side effects were mild to moderate and included monitorable gastrointestinal symptoms such as diarrhea, thrombocytopenia and hypertriglyceridaemia, and were manageable with dose adjustments. No severe or serious adverse events were directly related to the drug or resulted in study withdrawal (Mercuri et al., 2024).

## Future considerations for combination treatment

Now that givinostat is approved by the Food and Drug Administration (United States), there is an opportunity for combination with other approved treatments that induce production of partially functional dystrophin. The expectation is that the combination would have added benefit, as the dystrophin-restoring approaches rely on the presence of muscle and muscle quality. The micro-dystrophin gene therapy approach (delandistrogene moxeparvovec) relies on a muscle-specific promotor and thus the transgene will only be expressed in skeletal and cardiac muscle (Hoy, 2023). Furthermore, the micro-dystrophin is only partially functional and will slow down but not stop pathology. As such, a second treatment aiming to slow down muscle pathology should be beneficial. The exon skipping approach (eteplirsen, golodirsen, casimersen and viltolarsen) uses antisense oligonucleotides that target exons during pre-mRNA splicing of dystrophin transcripts (Aartsma-Rus, 2023). These transcripts are only produced in muscle tissue and not in fibrosis or adipose tissues. For exon skipping however, an added benefit of givinostat co-treatment is expected, as it has been shown that dystrophin transcript expression is reduced in patients with DMD due to chromatin remodeling. It is expected that givinostat treatment can increase dystrophin expression *per se*. Without exon skipping, this would not lead to dystrophin protein production; after exon skipping, it would. Indeed, in the *mdx* mouse model, treatment with mouse specific exon skipping compounds and givinostat resulted in increased levels of dystrophin transcripts and protein compared with exon skipping by itself (García-Rodríguez et al., 2020).

## Conclusion

DMD pathogenesis is complex and multifaceted. All currently-available dystrophin-restoring treatments restore only partially functional dystrophins that may slow down disease pathology, but the pathophysiological processes remain inevitable. HDACs have been shown to be hyperactive in patients with DMD and contribute to this pathology, therefore HDAC inhibition has arisen as a potential therapeutic option. Through its novel, multi-targeted mode of action, the HDAC inhibitor givinostat has demonstrated the potential to address the pathophysiological cascade of DMD by targeting key pathological events originated by the lack of dystrophin. The reduction of muscle degeneration is achieved by lowering inflammation in the muscle, reverting inhibition of myogenesis, promoting muscle regeneration, and decreasing fibrogenesis and adipogenesis in patients with DMD.

Givinostat is the first nonsteroidal treatment for DMD to be approved for use irrespective of the specific genetic variant underlying the disease and received its first approval for the treatment of DMD in patients ≥6 years old in March 2024 in the United States (Lamb, 2024). Ongoing clinical studies continue to evaluate the potential of HDAC inhibition in DMD and other disorders where elevated HDAC activity plays a role.

## Abbreviations

DMD, Duchenne muscular dystrophy; DAPC, dystrophin-associated glycoprotein protein complex; FAP, fibro-adipogenic progenitor; HDAC, histone deacetylase; MHCI, major histocompatibility complex class I; MCHII, major histocompatibility complex class II; miRNA, microribonucleic acid; MPO, myeloperoxidase; MuSC, muscle stem cell; NOS, nitric oxide synthase.
